# Insights into the schizophrenia and dental care: focusing on interaction between implant treatments and oxidative stress

**DOI:** 10.3389/fdmed.2025.1542913

**Published:** 2025-02-28

**Authors:** Ecaterina Burlui, Viorica Rarinca, Alin Ciobica, Vasile Burlui, Romeo Dobrin, Carmen Stadoleanu

**Affiliations:** ^1^ Doctoral School of Biology, Faculty of Biology, “Alexandru Ioan Cuza” University of Iași, Iasi, Romania; ^2^ “Ioan Haulica” Institute, Apollonia University, Iasi, Romania; ^3^ Doctoral School of Geosciences, Faculty of Geography and Geology, Alexandru Ioan Cuza University of Iasi, Iasi, Romania; ^4^ Faculty of Biology, “Alexandru Ioan Cuza” University of Iași, Iasi, Romania; ^5^ Center of Biomedical Research, Romanian Academy, Iasi, Romania; ^6^ Preclinical Department, “Apollonia” University of Iasi, Iasi, Romania; ^7^ Department of Psychiatry, “Grigore T. Popa” University of Medicine and Pharmacy, Iasi, Romania

**Keywords:** schizophrenia, dental care, oxidative stress, dental treatment, dental implants

## Abstract

Schizophrenia is a complex mental condition characterized by the deterioration of thought processes and inappropriate emotional responses. Compared to the general population, individuals diagnosed with schizophrenia have an increased risk of developing various dental diseases, including dental caries, periodontal disease, oral mucosal diseases, and conditions associated with oral resonance. In this context, we propose to establish links between schizophrenia and dental illness, emphasizing the importance of oxidative stress (OS) markers in schizophrenia, and also the role of dental treatments, particularly dental implants. This highlights the urgent need for an intervention program to improve oral health in patients with schizophrenia, including aspects such as prevention and prosthetic treatment. Dental implants can be a favorable option, offering good aesthetic and functional results for treated patients with schizophrenia. Additionally, a carefully developed surgical plan is essential, requiring collaboration between psychiatry, oral and maxillofacial surgery, anesthesiology, and prosthodontics departments.

## 1 Introduction

Schizophrenia is a chronic psychiatric illness with complex symptoms (1). According to Schultz et al., schizophrenia affects about 1% of the population worldwide (2). Patients with Schizophrenia are characterized by positive and negative symptoms. Although the notion as well as the limits of the diagnosis itself have expanded but some of these symptoms in literature remain the same including hallucination, delusion, social withdrawal and cognitive deficits (3).

Oral health is essential for daily activities, such as eating, smiling, and speaking (4). On the other hand, poor oral health can negatively impact overall health, self esteem, and quality of life (5). Individuals with psychotic disorders tend to have worse oral health outcomes compared to the general population, including higher rates of tooth decay and tooth loss (6, 7).

Oral care in patients with schizophrenia is a challenge for these patients. In most cases, patients do not take care of their health, do not brush their teeth, which is why they are prone to the appearance of tartar and gingival plaque. This unconsciousness about oral health later leads to the appearance of various dental diseases, such as caries, gingivitis and periodontitis (3).

The poor state of oral health in individuals with schizophrenia has been attributed to a range of factors, including poverty, lack of motivation, limited resources, imbalanced mental status, the side effects of antipsychotic medications, and the impact of coexisting systemic diseases such as diabetes, hypertension, and osteoporosis, which collectively diminish patients' ability to engage in effective selfcare (8). First-generation antipsychotics, including haloperidol, chlorpromazine, thioridazine, and trifluoperazine, are known to induce hyposalivation by inhibiting parasympathetic stimulation of the salivary glands. This reduction in saliva increases the risk of dental caries, especially root caries (9). Reduced salivation can lead to several side effects, including difficulty speaking, chewing disorders, inflammation of the mucous membranes (mucositis), oral Candida infections, and mucosal atrophy. Additionally, decreased saliva flow can lead to increased plaque buildup (10).

Fear and anxiety in patients with schizophrenia can manifest as premature tooth wear. Many individuals with schizophrenia exhibit parafunctional habits such as nail biting and lip biting. Additionally, bizarre behaviors leading to self mutilation, including autoextractions, glossectomy, and excoriation of gingival tissues with sharp fingernails, have been reported (11, 12). Studies indicate that oral health tends to deteriorate with increasing age and length of hospitalization, and it is generally worse among inpatients compared to outpatients (13, 14). Studies show that 88% of people diagnosed with schizophrenia have proven to be heavy tobacco abusers (15). The reasons for such a high frequency of heavy smoking prevalent in patients with schizophrenia are thought to be partly related to its enhanced stimulant effect. Smoking induces the release of dopamine in the brain, stimulating its activity and inhibiting its degradation (16). According to several authors, patients with schizophrenia do not always receive the best oral health treatment (17–20).

The liaison between psychiatrists and dentists is crucial, and developing consultation liaison models is essential for effectively treating patients with mental health issues. These models ensure integrated care, addressing both mental and oral health needs comprehensively.

Extreme empathy, patience, and tact are essential qualities for psychiatrists and dentists working together, especially when addressing the complex needs of patients with mental health conditions. Additionally, they must possess a thorough understanding of potential adverse reactions to commonly prescribed medications and be aware of any legislative issues that may impact treatment. This knowledge helps in managing patient care effectively and navigating potential challenges in a collaborative manner.

Hence, the proposed framework of Psychiatric Dental Consultation Liaison (PDCL) services is gaining success and popularity, offering a confident and structured approach to integrating mental health and oral care. This framework enhances collaboration between psychiatrists and dentists, leading to more comprehensive and effective patient care (21, 22).

The services, in addition to classic invasive and noninvasive procedures, include relaxation techniques and psychotherapy. The consultations carried out in the morning and of short duration, the quiet atmosphere and the direct communication and the calm explanations give the patients courage and the desire to return.

Oxidative stress is defined as a pathophysiological mechanism which appears at the moment of disturbance in the balance between the production of reactive oxygen species (ROS) and antioxidant defenses (23). The theory of oxidative stress, sometimes referred to as the “oxygen paradox”, that while oxygen is essential for aerobic life, excessive amounts of free radical metabolic byproducts are toxic (24, 25).

There are theories that oxidative stress mechanisms have been implicated in the pathogenesis of psychiatric disorders. The brain is considered particularly vulnerable to oxidative damage for several reasons (26). These include comparatively high oxygen utilization and hence generation of free radical byproducts, modest antioxidant defenses, lipid rich constitution that provides ready substrates for oxidation and the reducing potential of certain neurotransmitters. Additionally, the brain is also susceptible to secondary and self-perpetuating damage from oxidative cellular injury or necrosis, via? the neurotoxic effects of released excitatory amines (mainly glutamate) and iron, and the activated inflammatory response (27). Although the exact mechanism of schizophrenia is not known, it is assumed that at the basis of these pathologies is primarily the genetic factor, but also the nongenetic factors such as drug and alcohol abuse, stressful lifestyle, medications, prenatal and neonatal infections, complications during birth, etc. By inducing metabolic stress cellular, these factors appear to increase the possibility of oxidative stress and damage (28, 29).

There is ample evidence that a variety of environmental factors contribute to the dysregulation of gene expression caused by abnormal regulation of redox sensitive transcriptional factors, noncoding ribonucleic acids (RNAs), and epigenetic mechanisms (30).

**Figure 1:**
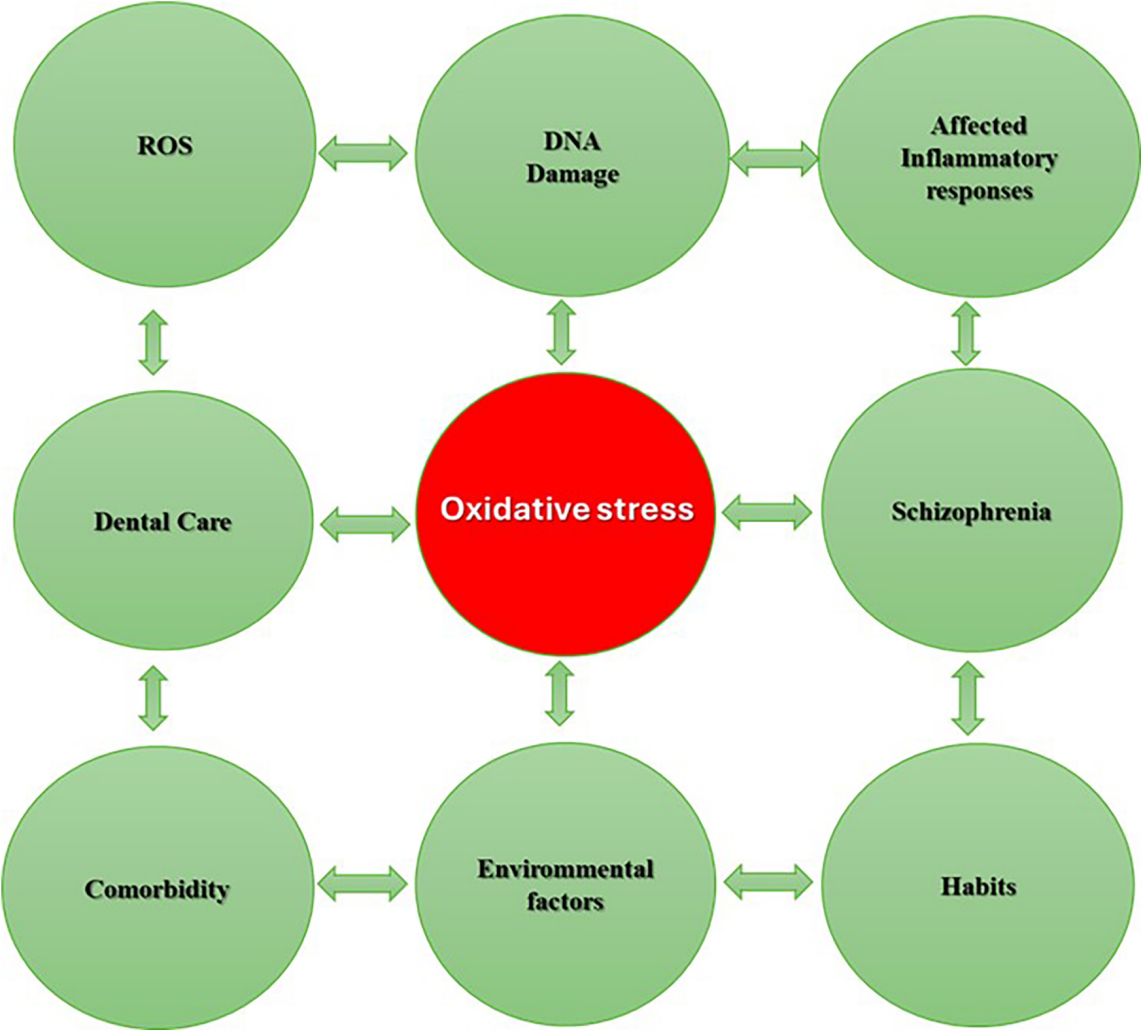
The connection between oxidative stress, dental care and schizophrenia.

Figure 1 illustrates the relationship between oxidative stress and various factors such as reactive oxygen species, DNA damage, inflammatory responses, schizophrenia, dental care, comorbidities, environmental factors, and habits. It's a representation of how oxidative stress can be central to general and oral health, especially in patients with schizophrenia.

## 2 Materials and methods

### 2.1 Search strategy

The current review was conducted following the Preferred Reporting Items for Systematic Review and MetaAnalysis (PRISMA) guidelines, employing several electronic databases (Science Direct, PubMed, and Google Scholar) to conduct a comprehensive and systematic search using the following search terms: ((schizophrenia[Title/Abstract]) AND (dental care[Title/Abstract])) AND ((schizophrenia[Title/Abstract]) AND (oxidative stress[Title/Abstract])) AND ((oxidative stress[Title/Abstract]) AND (dental care[Title/Abstract])) AND ((schizophrenia[Title/Abstract]) AND (dental implant[Title/Abstract])). The inclusion criteria focused on studies published up until inception of November 2024 in English, proposes to establish the connection between schizophrenia and dental disease, emphasizing the importance of oxidative stress markers in schizophrenia, and the implementation of dental treatments in patients diagnosed with schizophrenia, with a particular focus on dental implant.

### 2.2 Excluding criteria

We applied the following exclusion criteria: (1) letters, summaries, expert opinions, and comments; (2) conference abstracts, books, book chapters, and unpublished results; (3) non-English papers.

### 2.3 Data extraction

Out of the initial 1,327 reports collected through electronic search, 826 were omitted due to duplication, 156 were excluded based on article type, and an additional 209 were excluded as they comprised conference abstracts, books, book chapters, and unpublished results. Furthermore, 8 were excluded because they were not in English.

### 2.4 Data synthesis

**Figure 2:**
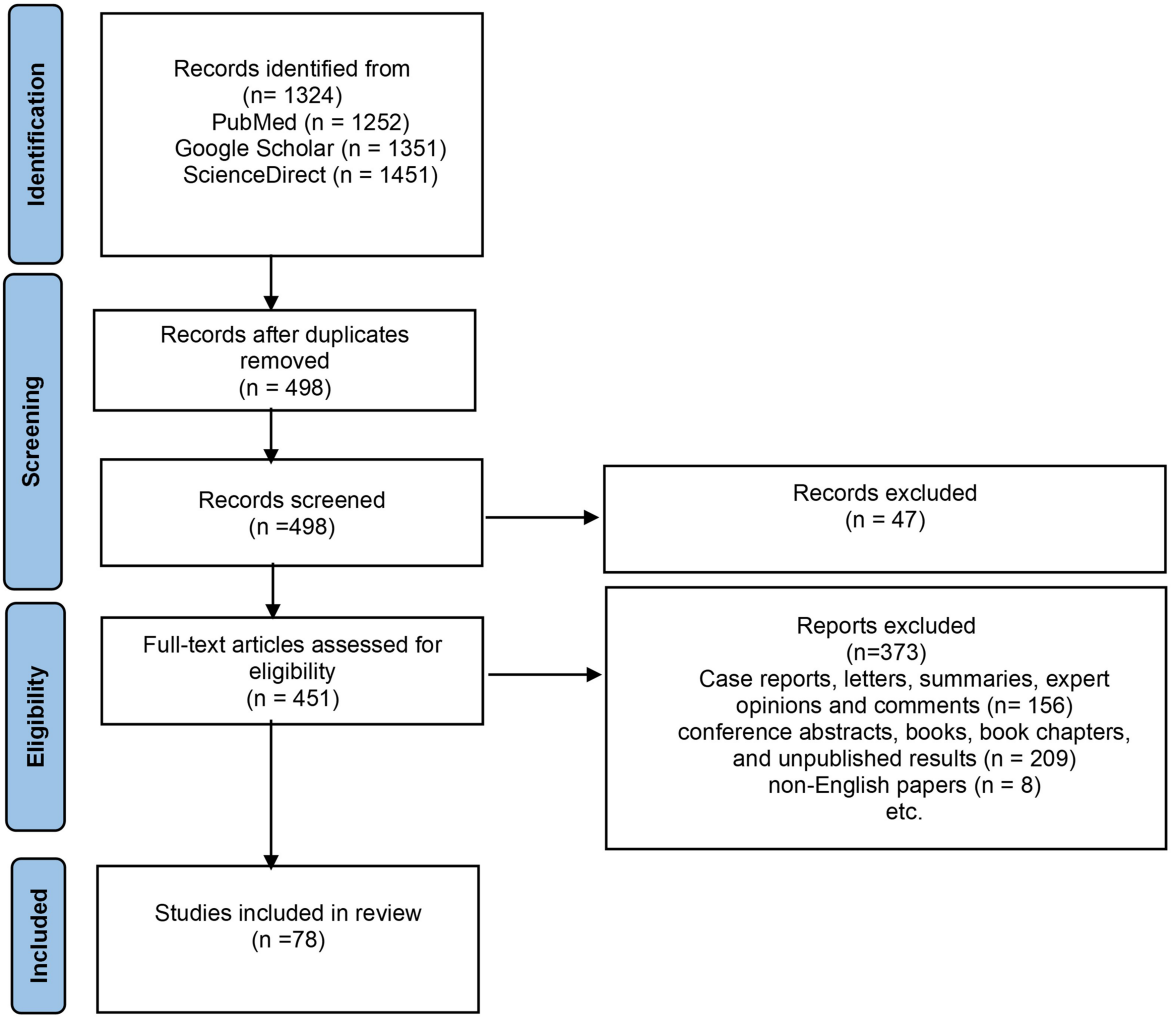
A flow diagram of the search strategy conducted (PRISMA flow of study selection process).

Finally, 81 articles were included in this review demonstrated in a diagram of the literature search and selection process (Figure 2). Due to the heterogeneity of the studies, a narrative synthesis was deemed the most suitable approach. The results are summarized in one chapter about dental implants and dental treatment in schizophrenic patients and in Table 1 that compresses the study's parameters and the results that have been reported in these cases. Then, the discussion chapter continues with 5 subsections, 4 of them present information found in literature about dental implants, dental implants and oxidative stress, oxidative stress and dental health, one about schizophrenia and oxidative stress and a final one with limitations.

**Table 1:** Analysis of studies on dental implants in schizophrenic patients.

Study	Parameters	Results	References
A man of 33 years old, diagnosed with Schizophrenia. Treatment: Haloperidol, oxcarbazepine, Olanzapine (Seroquel). Evaluation: multiple caries dental, waste rooting, lack lateral incisors. A consultation was carried out beforehand with a specialist psychiatrist and were not noted contraindications.	The treatment plan a including restoration tooth decay, fragment extractions root canals and prostheses supported on the implant a lateral incisors superiors bilaterally, from aesthetic reasons. After anesthesia local, first implant was placed in the upper right (Straumann Bone Level Ø 3.3 mm, length 10 mm). Two more weeks later, a second one implant was placed in the lateral position top left (Straumann Bone Level Ø 3.3 mm, length 12 mm).	Prosthesis (crown of the tooth) was placed after 3 months and both the patient as well as the family have been fully satisfied with results. There were routine checks performed at 6 months and 1 year after placement the implant of coronary prosthesis.	(31)
In June 2014, a man aged 62 years present for multiple carious lesions. Basic comorbidities they included schizophrenia and hypothyroidism. Medication: Levothyroxine Sodium (0.075 mg per day), Clozapine (400 mg per day), Lorazepam (0.5 mg per day), Propranolol (40 mg per day), Haloperidol (10 mg per day) and Risperidone (4 mg per day).	After the problem caries was solved, settled as a plan of extraction treatment teeth that cannot be also deal with the replacement missing teeth with dental implants. After it was realized general anesthesia, in there were two meetings extracted 16 teeth and roots and were immediately entered 10 implants, with addition of bone, in the missing areas.	The patient was continuously monitored and had professional cleanings performed at the Prosthetics Department. The dental implants were successfully placed, and the prosthetic teeth were positioned over the implants six months later. No accommodation problems or physical or psychological disturbances were reported, the patient being compliant and expressing satisfaction with the results.	(32)
A 55 years old lady suffering from schizophrenia visited Sujay's Dental Care complaining about tooth mobility, bad breath smelling. In addition, she was not able to chew properly.	After the achievement x-rays, it was set the diagnosis of periodontitis generalized, teeth remaining being very furniture, with plate bacterial and tartar. The treatment plan began with the extraction all teeth rest and rehabilitation complex, with 6 jaw implants and 5 implants at mandible. Welded between them with bar of titanium.	The intervention was successful, despite the complexity of the case. Based on the implants, a prosthesis with 14 teeth was made for the jaw and a prosthesis with 12 teeth in the mandible. Following the procedures, the patient was advised to schedule regular follow-ups every six months.	(33)

## 3 Results

Table 1 contains information about the cases we found in the literature about dental implants in patients with schizophrenia. In all cases patients are presented with multiple caries, required extractions, or generalized tooth damage. Therefore, dental implant treatment was chosen to ensure the necessary stability, functionality, and aesthetics.

In the fist case, after completing the treatment plan, including the placement of two implants for the missing lateral incisors, the results were highly positive, with good osteointegration. At the 6-month follow-up, the implants were fully osseointegrated, as confirmed by radiographic evaluation. The patient reported significant improvement in chewing efficiency and self-confidence due to the restored aesthetics. Castellanos-Cosano et al., report no signs of peri-implant inflammation or complications were observed during the monitoring period (31). The same good results were obtained in the second case, even though it was a more complex rehabilitation and as additional comorbidities the 62-year-old patient also had hypothyroidism, requiring extra caution in managing systemic risks.

Mild gingival inflammation was detected during early follow-ups but was resolved through professional scaling and enhanced oral hygiene practices. The patient reported better dietary habits and an improved sense of well-being, with no adverse reactions to the procedures or medications used (32).

In the third case, where it was necessary to extract all the remaining, but periodontal teeth, the treatment was a complete oral rehabilitation with 6 maxillary and 5 mandibular implants resulted in stable and functional prostheses (33). The patient expressed satisfaction with the restored ability to chew and improved aesthetics. However, she required regular motivation and guidance to maintain proper oral hygiene. Minor adjustments to the prostheses were necessary to address food impaction issues, but overall, the results were successful.

These studies highlight the significant advancements in dental treatments for individuals with schizophrenia. They demonstrate that promising outcomes can be achieved, particularly through the successful healing of dental implants. Such treatments not only improve oral health but also contribute positively to the mental and psychological well-being of patients, helping to address the stigma often associated with their condition.

## 4 Discussion

### 4.1 Dental implants on schizophrenic patients

The individuals with schizophrenia are generally prone to orofacial adverse effects such as xerostomia, sialorrhea and oral dyskinesia caused by psychotropic drugs (17). Healing after dental implant placement heavily depends on maintaining excellent oral hygiene. For individuals with schizophrenia, adhering to a rigorous oral hygiene routine may be particularly challenging due to factors such as cognitive impairments, side effects of medication, or lack of motivation (31).

Patients with schizophrenia may already have a higher degree of oral inflammation, either due to poor hygiene or due to systemic factors associated with the disease (34).

General inflammation and oxidative stress already present in the body can compress bone regeneration and tissue healing (35).

As important as medication is during treatment and surgery, so is antibacterial medication for afterwards. Also, the balance of the oral microbiota and the immune system play an important role in oral health. Any associated comorbidity, autoimmune diseases, a compromised immune system or an organ transplant (36). All of these aspects discussed have an interdisciplinary relationship and can influence the state of oral health. That being said, dentists are generally unwilling to invest in complex conservative or rehabilitative treatments because of the difficulty of treating patients with psychiatric illnesses (37).

### 4.2 Oxidative stress and dental implants

Dental implants are increasingly chosen as a preferred method for replacing missing natural teeth due to their notable success rates and numerous benefits.

Dental implant materials are typically composed of titanium (Ti) or Ti alloys. Upon exposure to oxygen, a titanium oxide (TiO_2_) layer forms on the surface of the implant. The integrity of this oxide layer is crucial to ensure proper osseointegration and prevent corrosion, thereby maintaining the stability of the bone implant interface (38).

After a dental implant is placed, an inflammatory healing response occurs, characterized by processes such as angiogenesis. Angiogenesis, the formation of new blood vessels, plays a crucial role in delivering cells and oxygen necessary for the successful integration of the implant with the surrounding bone (osseointegration) (35).

Tsarik et al., studied the effects of cathodic corrosion of titanium (Ti) on endothelial cells and found that it led to cytotoxicity and inhibition of cell proliferation. They identified ROS and hydrogen peroxide (H_2_O_2_) as byproducts of the corrosion process (39).

Typically, a baseline level of ROS exists before a dental implant is placed (40). The process of dental implantation itself inevitably generates additional ROS. While ROS play essential roles in cell signaling and normal metabolism, excessive oxidative stress can potentially cause damage to DNA, RNA, and proteins (41).

Thus, maintaining a balance in ROS levels during and after implant placement is crucial for minimizing oxidative damage and promoting successful integration of the implant with the surrounding tissues.

The sources of ROS in addition to dental materials are also relatively wide, mainly including food, cigarettes, alcohol, and drugs (42). As people increasingly prioritize dental health, various dental treatment procedures are gaining popularity. Consequently, the sources of ROS are more diverse and widespread than anticipated. Schizophrenia has the potential to worsen oral health by impairing the subject's ability to maintain oral hygiene. There is limited literature on how oral diseases manifest in individuals with schizophrenia (19).

Currently, the use of the implant in patients generally has a high, long term success rate of 90%–95% (43). However, implant treatment in disabled patients has a limited application due to the lack of compliance and oral care (44).

### 4.3 Oxidative stress and dental health

Oxidative stress underpins conditions such as diabetes, rheumatoid arthritis, nonalcoholic fatty liver disease, chronic inflammation, stroke, aging, various neurodegenerative diseases, and cancer (45–48). During recent years, there has been increasing research interest in the relationship between oxidative stress and oral health.

Free radicals, highly reactive molecules with unpaired electrons, can cause oxidative stress by damaging cellular components like deoxyribonucleic (DNA), proteins, and lipids. In the mouth, oxidative stress can contribute to conditions such as periodontal disease, oral cancers, lichen planus and dental caries (49).

Periodontitis is a serious gum infection that damages the soft tissue and destroys the bone that supports teeth. The fundamental causative factor is bacteria and the engagement in the destroying of the periodontium. Polymorphonuclear neutrophils (PMNs), often simply called neutrophils, are indeed crucial components of the innate immune response against bacterial pathogens. PMNs migrate to sites of inflammation in response to proinflammatory cytokines secreted as part of the immune response. Upon arrival, they utilize Nicotinamide adenine dinucleotide phosphate (NADPH), mononitrogen oxides (NOx), reactive oxygen species (ROS) and proteolytic enzymes, which are essential for combating bacterial pathogens and contributing to the inflammatory process (50).

Baltacioglu et al., investigated the serum and the saliva from patients with chronic (CP) and generalized aggressive periodontitis (GAgP). Salivary malondialdehyde (MDA) levels, as well as serum and salivary total oxidant status (TOS) and oxidative stress index (OSI) values, were found to be significantly higher in the periodontitis groups compared to the control group (51). Another study that includes patients with chronic periodontitis, detected that a overproduction of ROS by activated polymorphonuclear leukocytes in chronic inflammation may lead to premature oxidative damage of the mitochondrial DNA (mtDNA) (52). Numerous studies have explored the association between oxidative stress and oral cancer, which involves mechanistic pathways contributing to cancer initiation and progression. Overexpression of enzymes such as NOx, Cyclooxygenase (COX), Lipoxygenase (LOX), and nitric oxide synthase (NOS) serves as significant sources of ROS/reactive nitrogen species (RNS) in oral cancer (53–58). Oral cancer refers to cancers that develop in the mouth, including the lips, tongue, cheeks, gums, floor of the mouth, and roof of the mouth. ROS/RNS modulate the activity of a variety of transcription factors, including Apurinic/apyrimidinic endonuclease 1/redox effector factor 1 (APE1/Ref1), Hypoxia-Inducible Factor (HIF1α), Activator protein 1 (AP1), nuclear factor erythroid 2–related factor 2 (Nrf2), Nuclear factor-kappa B (NFκB), p53 gene, forkhead box transcription factors (FOXO), and *β*catenin (57). These factors are pivotal in regulating genes associated with cell proliferation, survival, and apoptosis, thereby exerting significant influence on cancer progression (57). Oral lichen planus (OLP) is a chronic inflammatory disease that affects the mucous membranes inside the mouth. It's characterized by white, lacy patches, redness, and sometimes painful sores. OLP is known for its relatively high prevalence, ranging from 0.5% to 2% of the population, depending on the region and demographic studied. Its chronic and recurrent nature makes it a significant concern for those affected. Additionally, there is a small but established potential risk of malignant transformation, particularly to oral squamous cell carcinoma (58–60).

In patients with Oral Lichen Planus (OLP), the study found that total antioxidant defense (TAA) was significantly lower in serum samples compared to healthy subjects (61). This suggests a decreased ability to combat oxidative stress systemically in OLP. Another study points to the possible function of oxidative stress in the etiopathogenesis of OLP. The mean levels of salivary MDA were significantly higher in the OLP group (62).

These findings highlight the presence of oxidative stress and impaired antioxidant defense mechanisms in OLP, potentially influencing its pathogenesis and suggesting avenues for therapeutic intervention aimed at restoring antioxidant balance.

Saliva indeed plays a crucial role in protecting the oral cavity and controlling dental caries. Its buffering effect helps maintain a stable pH level, which is essential for preventing acidic conditions that can lead to tooth decay. Additionally, saliva possesses antioxidant capacity, which helps neutralize free radicals. This equilibrium between antioxidants and free radicals is vital for overall oral health and contributes to the body's defense mechanisms (63, 64). Saliva acts as the primary defense against free radicals, utilizing various antioxidant mechanisms such as glutamate, ascorbic acid, uric acid, and melatonin. Additionally, it contains antioxidant enzymes like superoxide dismutase, catalase, and glutathione peroxidase. These components work together to counteract the detrimental effects of excessive reactive oxygen and nitrogen species in the oral cavity, maintaining a balance necessary for physiological function (65). Researchers observed a distinct pattern of salivary antioxidant responses to oxidative stress in children with caries, noting higher total antioxidant capacity (TAC) and superoxide dismutase levels in caries-free children, alongside lower levels of MDA (66, 67).

Dental caries is considered the most widespread chronic oral disease and is recognized as a significant oral health problem worldwide. It is an irreversible microbial disease affecting the hard tissues of the teeth, characterized by damage to both the inorganic and organic components of the tooth, leading to tooth decay and potential tooth loss (68).

### 4.4 Does oxidative stress play an important role in schizophrenia?

Currently, there are a large number of facts that indicate the development of pronounced oxidative stress in various diseases of the central nervous system. It is due to a combination of many important factors and characteristics of nervous tissue. The most significant of these is the high intensity of oxidative metabolism since 90% of the brain's energy needs are provided by aerobic processes (69).

In schizophrenia, there is a disruption of synaptic transmission and plasticity (70), including the disruption of N-methyl-D-aspartate receptor (NMDAR) activity, which is modulated by calcium ions (Ca^2+^). Additionally, Ca^2+^ ions activate neuronal nitric oxide synthase (nNOS), leading to the formation of nitric oxide (NO), carbonate (CO₃), and nitrite (NO₂) anions. These anions trigger neurodegeneration processes through the activation of heat shock protein 90 (Hsp90) and the induction of apoptosis (71).

Neurons have extremely weak antioxidant protection. Specifically, they contain 50 times less catalase compared to hepatocytes, making them more vulnerable to oxidative damage. The content of reduced glutathione (GSH) is approximately 50% lower in neurons compared to other cells. For instance, neurons have about 5 μM of GSH, whereas hepatocytes contain around 1,011 μM (72).

Numerous studies indicate a decreased concentration of reduced glutathione and an increased concentration of oxidized glutathione in various biological samples of schizophrenia patients. One of the studies conducted by Dariusz Juchnowicz revealed changes of catalase and glutathione peroxidase, which can be determining factors of schizophrenia (73). These findings have been observed in plasma (74), erythrocytes, neutrophils, platelets (73), cerebrospinal fluid (75), and in different postmortem brain regions (75–77). Although in patients with schizophrenia compared to controls, significant changes were observed in nitric oxide (NO) levels, but not in catalase (CAT) levels. However, alterations were noted in other antioxidant enzymes such as plasma glutathione peroxidase (GSHPx) and superoxide dismutase (SOD) (78).

In studies comparing neuroleptic-treated and untreated schizophrenic patients, both groups exhibited similar activities of SOD, which were approximately 60% higher than those found in normal, healthy individuals (30). This suggests that elevated SOD activity is a consistent feature in schizophrenia, regardless of neuroleptic treatment status.

### 4.5 Limitations

The limitations of the presented cases can be categorized in biological and physiological, reflecting the challenges and constraints associated with dental treatment for patients with schizophrenia.

The use of antipsychotics and other drugs (e.g., Haloperidol, Clozapine, or Risperidone) can cause xerostomia, which affects post-operative healing (79, 80). Also, they are known that hyposalivation can induce high risk of caries, periodontitis, which are a predisposing factor for a poor oral health (9).

There is also a limitation regarding the long-term verification of the patient. Even if the primary integration has been seen, we cannot know if the patient will comply with all the indications for a long period of time. The limitations of these cases reflect the complexity of managing patients with schizophrenia, emphasizing the need for rigorous planning, effective communication among specialists, and long-term monitoring to ensure treatment success.

## 5 Future directions

In recent years, research interest in the relationship between oxidative stress, inflammation, and oral health has grown significantly. However, there is still limited literature on how oral diseases and inflammation manifest in individuals with schizophrenia (19). Meanwhile, dental implants have shown high long-term success rates in most patients (39). Recently there are studies that show how choosing implant materials that reduce the risk of oxidative stress, such as implants specially treated to prevent the accumulation of ROS (10).

These clinical cases are important for both scientists and people with neuropsychiatric conditions, as they provide practical and relevant examples for the management of patients with complex medical conditions, such as schizophrenia, and demonstrate how dental treatments can be tailored to the patient's overall health status.

The essence of these clinical cases encompasses both the multidisciplinary approach and the fact that complex dental procedures can be performed with good management of systemic complications. They can serve as a foundation for the development of new protocols to balance risks, inflammation, and medical education among patients with neuropsychiatric diseases such as schizophrenia.

## 6 Conclusion

While it is well accepted that OS contributes significantly to the pathogenesis of schizophrenia, there is no consensus on whether OS is the primary cause of the disease or if it occurs as a secondary effect influenced by environmental factors or long-term treatments. Individuals with schizophrenia face a heightened risk of oral health issues, including dental caries, periodontal disease, and oral mucosal conditions, compared to the general population. Although we are in the stage of literature, in light of these findings, therapeutic interventions aimed at restoring oxidative balance, such as antioxidant therapies, hold promise for preventing and managing oxidative stress related oral diseases. A better understanding of the mechanisms linking oxidative stress to oral health can guide the development of targeted strategies to improve patient outcomes and overall oral health. Patients with schizophrenia face unique challenges in implant therapy due to impaired oral hygiene, limited compliance, and the added risk of oxidative stress. Despite the increasing use of dental implants, there is a significant lack of research on their outcomes in individuals with neuropsychiatric disorders, particularly schizophrenia. This gap underscores the need for tailored approaches, multidisciplinary care, and targeted research to address the specific needs of this population.

Fostering collaboration between dental professionals, psychiatrists, and caregivers, while incorporating strategies to mitigate oxidative stress, is essential for improving oral health outcomes and expanding access to implant therapy for patients with schizophrenia and other neuropsychiatric conditions.

## References

[1] Bitanihirwe BKY Woo TUW. Oxidative stress in schizophrenia: an integrated approach. Neurosci Biobehav Rev. (2011) 35:878–93. 10.1016/j.neubiorev.2010.10.008 20974172 PMC3021756

[2] Schultz SH North SW Shields CG. Schizophrenia: a review. Am Fam Physician. (2007) 75:1821–9. 17619525

[3] Yang M Chen P He MX Lu M Wang HM Soares JC Poor oral health in patients with schizophrenia: a systematic review and meta-analysis. Schizophr Res. (2018) 201:3–9. 10.1016/j.schres.2018.04.031 29759350

[4] Slade GD. Communify dentistiy and oral epidemiology derivation and validation of a short-form oral health impact profile. Community Detit Oral Epidemiol. (1997) 25:284–90. 10.1111/j.1600-0528.1997.tb00941.x 9332805

[5] Kisely S. No mental health without oral health. Can J Psychiatry. (2016) 61:277–82. 10.1177/0706743716632523 27254802 PMC4841282

[6] Kang J Wu J Aggarwal V Shiers D Doran T Palmier-Claus J. Oral Health Inequality in People with Severe Mental Illness: A Cross-Sectional Study Using National Health and Nutrition Examination Survey 1999–2016. *medRxiv*. (2021).

[7] Mehrabanian M Ahari UZ Fathi A Parizi MM. Comparison of dental health Status in schizophrenic patients with healthy individuals. Clin Schizophr Relat Psychoses. (2021) 15:1–5. 10.3371/CSRP.MMUA.102821

[8] Chu KY Yang NP Chou P Chiu HJ Chi LY. Factors associated with dental caries among institutionalized residents with schizophrenia in Taiwan: a cross-sectional study. BMC Public Health. (2010) 10:482. 10.1186/1471-2458-10-482 20707911 PMC2931468

[9] McGorry P. Royal Australian and New Zealand college of psychiatrists clinical practice guidelines for the treatment of schizophrenia and related disorders. Aust N Z J Psychiatry. (2005) 39:410–72. 10.1177/0004867416641195 27106681

[10] Greenberg M Glick M Ship J. Burket’s Oral Medicine. 11th ed. (2008).

[11] Tenzer JA Orozco H. Traumatic glossectomy. Report of a case. Oral Surg Oral Med Oral Pathol. (1970) 30:182–4. 10.1016/0030-4220(70)90358-0 5270919

[12] Mester R. The psychodynamics of the dental pathology of chronic schizophrenic patients. Isr J Psychiatry Relat Sci. (1982) 19:255–61. 7184896

[13] Kenkre AM Spadigam AE. Oral health and treatment needs in institutionalized psychiatric patients in India. Indian J Dent Res. (2000) 11:5–11. 11307250

[14] Friedlander AH Marder SR. The psychopathology, medical management and dental implications of schizophrenia. J Am Dent Assoc. (2002) 133:603–10. 10.14219/jada.archive.2002.0236 12036166

[15] Hughes JR Hatsukami DK Mitchell JE Dahlgren LA. Prevalence of smoking among psychiatric outpatients. Am J Psychiatry. (1986) 143:993–7. 10.1176/ajp.143.8.993 3487983

[16] Malhotra R Kapoor A Grover V Kaushal S. Nicotine and periodontal tissues. J Indian Soc Periodontol. (2010) 14:72–9. 10.4103/0972-124X.65442 20922084 PMC2933534

[17] Kilbourne AM Horvitz-Lennon M Post EP McCarthy JF Cruz M Welsh D Oral health in veterans affairs patients diagnosed with serious mental illness. J Public Health Dent. (2007) 67:42–8. 10.1111/j.1752-7325.2007.00007.x 17436978

[18] Kiyak HA Reichmuth M. Barriers to and enablers of older Adults’ use of dental services. J Dent Educ. (2005) 69:975–86. 10.1002/j.0022-0337.2005.69.9.tb03994.x 16141083

[19] Gupta S P PK Gupta R. Necessity of oral health intervention in schizophrenic patients—a review. Nepal J Epidemiol. (2017) 6:605–12. 10.3126/nje.v6i4.17254 PMC5506385 28804672

[20] Dickerson FB McNary SW Brown CH Kreyenbuhl J Goldberg RW Dixon LB. Somatic healthcare utilization among adults with serious mental illness who are receiving community psychiatric services. Med Care. (2003) 41:560–70. 10.1097/00005650-200304000-00011 12665719

[21] Saraiva S Bachmann M Andrade M Liria A. Bridging the mental health treatment gap: effects of a collaborative care intervention (matrix support) in the detection and treatment of mental disorders in a Brazilian city. Fam Med Community Health. (2020) 8. 10.1136/fmch-2019-000263 32958519 PMC7507894

[22] Šarac Z Zovko R Curlin M Filakovi P. Dental medicine and psychiatry: the need for collaboration and bridging the professional gap. Psychiatr Danub. (2020) 32:151–8. 10.24869/psyd.2020.151 32796779

[23] Chaudhary P Janmeda P Docea AO Yeskaliyeva B Abdull Razis AF Modu B Oxidative stress, free radicals and antioxidants: potential crosstalk in the pathophysiology of human diseases. Front Chem. (2023) 11:1158198. 10.3389/fchem.2023.1158198 37234200 PMC10206224

[24] Davies KJ. Oxidative stress: the paradox of aerobic life. Biochem Soc Symp. (1995) 61:1–31. 10.1042/bss0610001 8660387

[25] Betteridge DJ. What is oxidative stress? Proceedings of the Metabolism: Clinical and Experimental. Vol. 49 (2000). 10.1016/s0026-0495(00)80077-3 10693912

[26] Ng F Berk M Dean O Bush AI. Oxidative stress in psychiatric disorders: evidence base and therapeutic implications. Int J Neuropsychopharmacol. (2008) 11:851–76. 10.1017/S1461145707008401 18205981

[27] Halliwell B. Oxidative stress and neurodegeneration: where are we now? J Neurochem. (2006) 97:1634–58. 10.1111/j.1471-4159.2006.03907.x 16805774

[28] Mahadik SP Pillai A Joshi S Foster A. Prevention of oxidative stress-mediated neuropathology and improved clinical outcome by adjunctive use of a combination of antioxidants and Omega-3 fatty acids in schizophrenia. Int Rev Psychiatry. (2006) 18:119–31. 10.1080/09540260600581993 16777666

[29] Ranjekar PK Hinge A Hegde MV Ghate M Kale A Sitasawad S Decreased antioxidant enzymes and membrane essential polyunsaturated fatty acids in schizophrenic and bipolar mood disorder patients. Psychiatry Res. (2003) 121:109–22. 10.1016/S0165-1781(03)00220-8 14656446

[30] Ermakov EA Dmitrieva EM Parshukova DA Kazantseva DV Vasilieva AR Smirnova LP. Oxidative stress-related mechanisms in schizophrenia pathogenesis and new treatment perspectives. Oxid Med Cell Longev. (2021) 2021:8881770. 10.1155/2021/8881770 33552387 PMC7847339

[31] Castellanos-Cosano L Corcuera-Flores JR Mesa-Cabrera M Cabrera-Domínguez J Torres-Lagares D Machuca-Portillo G. Dental implants placement in paranoid squizofrenic patient with obsessive-compulsive disorder: a case report. J Clin Exp Dent. (2017) 9:e1371–4. 10.4317/jced.54356 29302292 PMC5741853

[32] Choi Y Kim H Rhee S-H Ryoo S-H Karm M-H Seo K-S Multiple implant therapy with multiple inductions of general anesthesia in non-compliant patients with schizophrenia: a case report. J Dent Anesth Pain Med. (2019) 19:239–44. 10.17245/jdapm.2019.19.4.239 31501783 PMC6726886

[33] Implant supported overdenture. Sujay\’s Dental Care. Available online at: https://www.sujaysdentalcare.com/smile-story-of-the-week/dental-implants-case-smile-story- (accessed August 15, 2024)

[34] Santhosh Kumar S Cantillo R Ye D. The relationship between oral health and schizophrenia in advanced age—a narrative review in the context of the current literature. J Clin Med. (2023) 12:6496. 10.3390/jcm12206496 37892634 PMC10607055

[35] Esquivel-Chirino C Gómez-Landeros JC Carabantes-Campos EP Carmona-Ruiz D Valero-Princet Y Márquez-Correa C The impact of oxidative stress on dental implants. Eur J Dent Oral Heal. (2021) 2:1–8. 10.24018/ejdent.2021.2.1.37

[36] Weinberg MA Segelnick SL Kay LB Nair V. Medical and dental standardization for solid organ transplant recipients. N Y State Dent J. (2013) 79:35–40. 24600763

[37] Zusman SP Ponizovsky AM Dekel D Masarwa AES Ramon T Natapov L An assessment of the dental health of chronic institutionalized patients with psychiatric disease in Israel. Spec Care Dent. (2010) 30:35–40. 10.1111/j.1754-4505.2009.00118.x 20051070

[38] Suárez-López del Amo F Garaicoa-Pazmiño C Fretwurst T Castilho RM Squarize CH. Dental implants-associated release of Titanium particles: a systematic review. Clin Oral Implants Res. (2018) 29:1085–100. 10.1111/clr.13372 30280418

[39] Tsaryk R Kalbacova M Hempel U Scharnweber D Unger RE Dieter P Response of human endothelial cells to oxidative stress on Ti6Al4V alloy. Biomaterials. (2007) 28:806–13. 10.1016/j.biomaterials.2006.09.033 17049373

[40] Mouthuy PA Snelling SJB Dakin SG Milković L Gašparović AČ Carr AJ Biocompatibility of implantable materials: an oxidative stress viewpoint. Biomaterials. (2016) 109:10789–1698. 10.1016/j.biomaterials.2016.09.010 27669498

[41] Yanez M Blanchette J Jabbarzadeh E. Modulation of inflammatory response to implanted biomaterials using natural compounds. Curr Pharm Des. (2017) 23:6347–57. 10.2174/1381612823666170510124348 28521709 PMC5681444

[42] Ahmadi-Motamayel F Goodarzi MT Hendi SS Kasraei S Moghimbeigi A. Total antioxidant capacity of saliva and dental caries. Med Oral Patol Oral Cir Bucal. (2013) 18:e553–6. 10.4317/medoral.18762 23524431 PMC3731080

[43] Arvidson K Bystedt H Frykholm A von Konow L Lothigius E. A 3-year clinical study of astra dental implants in the treatment of edentulous mandibles. Int J Oral Maxillofac Implants. (1992) 7:321–9. 1289257

[44] Gabre P Martinsson T Gahnberg L. Longitudinal study of dental caries, tooth mortality and interproximal bone loss in adults with intellectual disability. Eur J Oral Sci. (2001) 109:20–6. 10.1034/j.1600-0722.2001.00965.x 11330930

[45] Fridovich I. Fundamental aspects of reactive oxygen species, or what’s the matter with oxygen? Proceedings of the Annals of the New York Academy of Sciences. Vol. 893 (1999). 10.1111/j.1749-6632.1999.tb07814.x 10672226

[46] Fang YZ Yang S Wu G. Free radicals, antioxidants, and nutrition. Nutrition. (2002) 18:872–9. 10.1016/S0899-9007(02)00916-4 12361782

[47] Gentric G Maillet V Paradis V Couton D L’Hermitte A Panasyuk G Oxidative stress promotes pathologic polyploidization in nonalcoholic fatty liver disease. J Clin Invest. (2015) 125:981–92. 10.1172/JCI73957 25621497 PMC4362240

[48] Pisoschi AM Pop A. The role of antioxidants in the chemistry of oxidative stress: a review. Eur J Med Chem. (2015) 97:55–74. 10.1016/j.ejmech.2015.04.040 25942353

[49] Jin LJ Lamster IB Greenspan JS Pitts NB Scully C Warnakulasuriya S. Global burden of oral diseases: emerging concepts, management and interplay with systemic health. Oral Dis. (2016) 22:609–19. 10.1111/odi.12428 26704694

[50] Waddington RJ Moseley R Embery G. Reactive oxygen Species: a potential role in the pathogenesis of periodontal diseases. Oral Dis. (2000) 6(3):138–51. 10.1111/j.1601-0825.2000.tb00325.x 10822357

[51] Baltacıoğlu E Yuva P Aydın G Alver A Kahraman C Karabulut E Lipid peroxidation levels and total oxidant/antioxidant status in serum and saliva from patients with chronic and aggressive periodontitis. Oxidative stress index: a new biomarker for periodontal disease? J Periodontol. (2014) 85:1432–41. 10.1902/jop.2014.130654 24635543

[52] Çanakçı CF Tatar A Çanakçı V Cicek Y Oztas S Orbak R. New evidence of premature oxidative DNA damage: mitochondrial DNA deletion in gingival tissue of patients with periodontitis. J Periodontol. (2006) 77:1894–900. 10.1902/jop.2006.060108 17076616

[53] Hezel MP Weitzberg E. The oral microbiome and nitric oxide homoeostasis. Oral Dis. (2015) 21:7–16. 10.1111/odi.12157 23837897

[54] Connelly ST Macabeo-Ong M Dekker N Jordan RCK Schmidt BL. Increased nitric oxide levels and INOS over-expression in oral squamous cell carcinoma. Oral Oncol. (2005) 41:261–7. 10.1016/j.oraloncology.2004.09.007 15743688

[55] Peymanfar Y Mahjour F Shrestha N de la Cueva A Chen Y Huang S The lysyl oxidase G473A polymorphism exacerbates oral cancer development in humans and mice. Int J Mol Sci. (2023) 24:9407. 10.3390/ijms24119407 37298359 PMC10254048

[56] McCormick DL Phillips JM Horn TL Johnson WD Steele VE Lubet RA. Overexpression of cyclooxygenase-2 in rat oral cancers and prevention of oral carcinogenesis in rats by selective and nonselective COX inhibitors. Cancer Prev Res. (2010) 3:73–81. 10.1158/1940-6207.CAPR-09-0151 PMC2804934 20051374

[57] Gào X Schöttker B. Reduction-oxidation pathways involved in cancer development: a systematic review of literature reviews. Oncotarget. (2017) 8:51888–906. 10.18632/oncotarget.17128 28881698 PMC5584299

[58] Roopashree MR Gondhalekar RV Shashikanth MC George J Thippeswamy SH Shukla A. Pathogenesis of oral lichen planus—a review. J Oral Pathol Med. (2010) 39:729–34. 10.1111/j.1600-0714.2010.00946.x 20923445

[59] Lodi G Carrozzo M Furness S Thongprasom K. Interventions for treating oral lichen planus: a systematic review. Br J Dermatol. (2012) 166:938–47. 10.1111/j.1365-2133.2012.10821.x 22242640

[60] van der Meij EH Mast H van der Waal I. The possible premalignant character of oral lichen planus and oral lichenoid lesions: a prospective five-year follow-up study of 192 patients. Oral Oncol. (2007) 43:742–8. 17112770 10.1016/j.oraloncology.2006.09.006

[61] Ergun S Troşala ŞC Warnakulasuriya S Özel S Önal AE Ofluoǧlu D Evaluation of oxidative stress and antioxidant profile in patients with oral lichen planus. J Oral Pathol Med. (2011) 40:286–93. 10.1111/j.1600-0714.2010.00955.x 21039889

[62] Lopez-Jornet P Martinez-Canovas A Pons-Fuster A. Salivary biomarkers of oxidative stress and quality of life in patients with oral lichen planus. Geriatr Gerontol Int. (2014) 14:654–9. 10.1111/ggi.12153 24205825

[63] Buzalaf MAR Hannas AR Kato MT. Saliva and dental erosion. J Appl Oral Sci. (2012) 20:493–502. 10.1590/s1678-77572012000500001 23138733 PMC3881791

[64] Lee YH Wong DT. Saliva: an emerging biofluid for early detection of diseases. Am J Dent. (2009) 22:241–8. 19824562 PMC2860957

[65] Tóthová L Kamodyová N Červenka T Celec P. Salivary markers of oxidative stress in oral diseases. Front Cell Infect Microbiol. (2015) 5:73. 10.3389/fcimb.2015.00073 26539412 PMC4611854

[66] Araujo HC Nakamune ACMS Garcia WG Pessan JP Antoniali C. Carious lesion severity induces higher antioxidant system activity and consequently reduces oxidative damage in children’s saliva. Oxid Med Cell Longev. (2020) 2020:3695683. 10.1155/2020/3695683 32089767 PMC7008261

[67] Da Silva PV Troiano JA Nakamune ACMS Pessan JP Antoniali C. Increased activity of the antioxidants systems modulate the oxidative stress in saliva of toddlers with early childhood caries. Arch Oral Biol. (2016) 70:62–6. 10.1016/j.archoralbio.2016.06.003 27328152

[68] Li G Shofer JB Rhew IC Kukull WA Peskind ER McCormick W Age-varying association between statin use and incident Alzheimer’s disease. J Am Geriatr Soc. (2010) 58:1311–7. 10.1111/j.1532-5415.2010.02906.x 20533968 PMC3176730

[69] Lohr JB Browning JA. Free radical involvement in neuropsychiatric illnesses. Proceedings of the Psychopharmacology Bulletin. Vol. 31 (1995). 7675980

[70] Pocklington AJ O’Donovan M Owen MJ. The synapse in schizophrenia. Eur J Neurosci. (2014) 39:1059–67. 10.1111/ejn.12489 24712986

[71] Franco MC Ye Y Refakis CA Feldman JL Stokes AL Basso M Nitration of Hsp90 induces cell death. Proc Natl Acad Sci U S A. (2013) 110:E1102–11. 10.1073/pnas.1215177110 23487751 PMC3607042

[72] Dwivedi D Megha K Mishra R Mandal PK. Glutathione in brain: overview of its conformations, functions, biochemical characteristics, quantitation and potential therapeutic role in brain disorders. Neurochem Res. (2020) 45:1461–80. 10.1007/s11064-020-03030-1 32297027

[73] Flatow J Buckley P Miller BJ. Meta-analysis of oxidative stress in schizophrenia. Biol Psychiatry. (2013) 74:400–9. 10.1016/j.biopsych.2013.03.018 23683390 PMC4018767

[74] Raffa M Atig F Mhalla A Kerkeni A Mechri A. Decreased glutathione levels and impaired antioxidant enzyme activities in drug-naive first-episode schizophrenic patients. BMC Psychiatry. (2011) 11:124. 10.1186/1471-244X-11-124 21810251 PMC3161936

[75] Do KQ Trabesinger AH Kirsten-Krüger M Lauer CJ Dydak U Hell D Schizophrenia: glutathione deficit in cerebrospinal fluid and prefrontal cortex *in vivo*. Eur J Neurosci. (2000) 12:3721–8. 10.1046/j.1460-9568.2000.00229.x 11029642

[76] Yao JK Leonard S Reddy R. Altered glutathione redox state in schizophrenia. Dis Markers. (2006) 22:83–93. 10.1155/2006/248387 16410648 PMC3850558

[77] Kumar J Liddle EB Fernandes CC Palaniyappan L Hall EL Robson SE Glutathione and glutamate in schizophrenia: a 7T MRS study. Mol Psychiatry. (2020) 25:1703. 10.1038/s41380-018-0104-7 PMC7156342 29934548

[78] Zhang M Zhao ZM He L Wan CL. A meta-analysis of oxidative stress markers in schizophrenia. Sci China Life Sci. (2010) 53:112–24. 10.1007/s11427-010-0013-8 20596963

[79] Tang WK Sun FCS Ungvari GS O’Donnell D. Oral health of psychiatric in-patients in Hong Kong. Int J Soc Psychiatry. (2004) 50:186–91. 10.1177/0020764004043134 15293435

[80] Kilian R Becker T Krüger K Schmid S Frasch K. Health behavior in psychiatric in-patients compared with a German general population sample. Acta Psychiatr Scand. (2006) 114:242–8. 10.1111/j.1600-0447.2006.00850.x 16968361

[81] Qi F Huang H Wang M Rong W Wang J. Applications of antioxidants in dental procedures. Antioxidants. (2022) 11:2492. 10.3390/antiox11122492 36552699 PMC9774737

